# MoSIoT: Modeling and Simulating IoT Healthcare-Monitoring Systems for People with Disabilities

**DOI:** 10.3390/ijerph18126357

**Published:** 2021-06-11

**Authors:** Santiago Meliá, Shahabadin Nasabeh, Sergio Luján-Mora, Cristina Cachero

**Affiliations:** Departamento de Lenguajes y Sistemas Informáticos, Universidad de Alicante, Carretera de San Vicente s/n, 03690 San Vicente del Raspeig, Alicante, Spain; nss24@alu.ua.es (S.N.); sergio.lujan@ua.es (S.L.-M.); ccc@ua.es (C.C.)

**Keywords:** Internet of Things, healthcare-monitoring system, disabilities, simulator, model-driven engineering, Alzheimer’s, FHIR, trigger–action programming, machine-learning MDE, Azure IoT Central

## Abstract

The need to remotely monitor people with disabilities has increased due to growth in their number in recent years. The democratization of Internet of Things (IoT) devices facilitates the implementation of healthcare-monitoring systems (HMSs) that are capable of supporting disabilities and diseases. However, to achieve their full potential, these devices must efficiently address the customization demanded by different IoT HMS scenarios. This work introduces a new approach, called Modeling Scenarios of Internet of Things (MoSIoT), which allows healthcare experts to model and simulate IoT HMS scenarios defined for different disabilities and diseases. MoSIoT comprises a set of models based on the model-driven engineering (MDE) paradigm, which first allows simulation of a complete IoT HMS scenario, followed by generation of a final IoT system. In the current study, we used a real scenario defined by a recognized medical publication for a patient with Alzheimer’s disease to validate this proposal. Furthermore, we present an implementation based on an enterprise cloud architecture that provides the simulation data to a commercial IoT hub, such as Azure IoT Central.

## 1. Introduction

Recently, the World Health Organization (WHO) [1] estimated that about one billion people have some form of disability (15% of the world’s population). One of the main problems identified by the WHO is that medical institutions should address the physical barriers that may hinder the healthcare and monitoring of patients with disabilities. Therefore, it is necessary to implement healthcare-monitoring systems (HMSs) devoted to monitoring their vital signs, with a set of different devices appropriate for each disability.

Furthermore, as a result of the increased democratization and reduced cost of Internet of Things (IoT) devices, technology has become more accessible to people with disabilities, with the aim of improving their independence and quality of life. However, monitoring people with disabilities is not a simple task: (i) each IoT HMS should consider a specific scenario that allows two-way communication between the person with a disability and the system; (ii) the devices must be suitable for collecting patients’ vital signs; (iii) the telemetry generated must be relevant to physicians; and (iv) the actions to be taken must be case-specific.

Numerous proposals have addressed the monitoring of people with disabilities [2,3,4,5]; however, these share three main drawbacks. First, to the best of our knowledge, none have attempted to address the customization needed for each IoT HMS scenario. To address this issue, the inclusion of personalization mechanisms in the proposals would facilitate the representation of the variability associated with the monitoring of people with disabilities.

In addition, the final implementation of these IoT HMSs often fails to acknowledge that healthcare experts need to undertake appropriate monitoring of patients [6]. One solution to this problem is that the HMS allows experts to take part in the configuration of the system. This can be achieved by raising the level of abstraction at which this HMS configuration takes place, and by providing a simplified configuration model that is closer to the expert’s domain.

Finally, the implementation cost of these systems is high. To address this issue, the IoT community [7] recognizes simulators as a useful mechanism to identify risk factors and evaluate these complex systems in advance.

In this work, we propose a new approach, called Modeling Scenarios of Internet of Things (MoSIoT), which, based on the model-driven engineering (MDE) [8] paradigm, can address these three previous challenges by: (i) providing a simple communication language that allows domain experts, in this case, healthcare workers, to configure an IoT HMS scenario; (ii) possessing the customization capability to modify and introduce new elements to the IoT HMS scenario; and (iii) establishing an enterprise cloud architecture that allows simulating the scenario in a real situation.

It is well known that the MDE paradigm establishes the use of models as central elements. These models are domain-specific languages that raise the level of abstraction, thus facilitating the communication between the application stakeholders. In addition, MDE provides an automation process based on model-to-model and model-to-text transformations, which reduce the time required for deployment to the final solution.

MoSIoT applies an IoT architecture reference model [9] that is specialized for IoT HMS systems for people with disabilities, and comprises two elements: (i) a domain model, in which a domain expert specifies a knowledge base that gathers the invariant information of most recognized standards relating to three fields, namely, accessibility, the IoT systems, and healthcare; and (ii) a scenario model, which allows a medical expert to introduce the static and dynamic behavior of the entities specific to a concrete IoT HMS scenario of patients with disabilities. The two models are connected, allowing the domain model’s invariant concepts to feed the variable entities defined in each of the scenarios.

**Figure 1:**
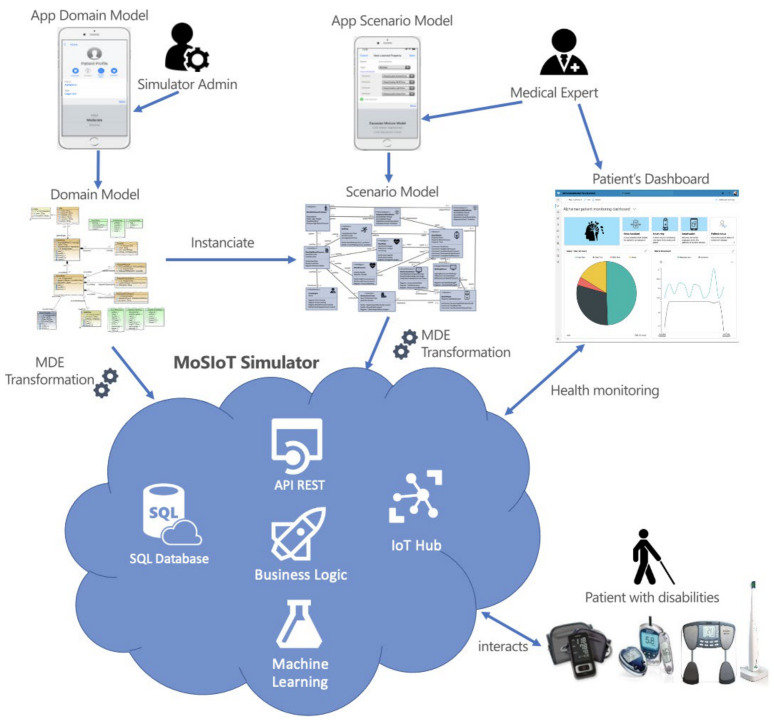
An overview of the MoSIoT framework.

As shown in Figure 1, the MoSIoT framework applies MDE to generate from these MoSIoT models an enterprise cloud architecture based on a set of Web services with a secure API REST interface, a transactional business logic, and database persistence. The architecture’s central component is the MoSIoT simulator, which manages the domain model information and contains the code generator to obtain each IoT HMS scenario. Each scenario contains specific business logic and the prediction module that applies machine learning (ML) to provide data to the IoT Hub. This solution uses Azure IoT Central [10], which has a powerful IoT accelerator that provides an API REST to send the IoT telemetries for presentation to the medical expert in a Web patient’s dashboard. This MoSIoT framework allows the domain and scenario models to be populated from Web or mobile apps, making the framework more usable and accessible by non-technical users, such as medical experts, caregivers, or related people.

This paper presents a case study for people with cognitive and physical disabilities caused by Alzheimer’s disease. Based on recent medical studies, we validated our approach, representing this real IoT HMS scenario covering the follow-up recommendations for these patients [11]. The case study validates the scenario model’s usefulness to simulate a scenario with the devices, vital signs, and accessible modes based on relevant medical literature on Alzheimer’s patients and cognitive disabilities.

This paper is structured as follows: Section 2 describes the previous work on IoT in healthcare-monitoring systems, IoT simulators, and MDE for IoT. Section 3 introduces the MoSIoT framework, presenting the software architecture model and the reference models of the MoSIoT framework. Section 4 presents a case study in which this approach was applied for Alzheimer’s disease (AD) patients. Section 5 explains the challenges to the validation of the proposal, and finally, Section 6 draws the conclusions of the paper and defines the main lines of future work.

## 2. Background

To contextualize the contribution of this work, this section focuses on introducing the existing approaches aligned to each of the goals of this research: (i) the existing solutions applied to healthcare-monitoring and assisted-living systems for caring for people with special needs; (ii) the range of IoT simulators currently available for HMS solutions; and (iii) the application of model-driven approaches in the development of IoT systems.

### 2.1. The Existing HMS Solutions for People with Disabilities

Concern regarding the increase in the number of people with disabilities is not new. Proposals for a technological vision aimed at improving the quality of life and increasing the independency of people with special needs have been made for a number of years [2,3]. These approaches appeared under the term “smart houses” (SHs), which apply a model that is adapted to the user’s needs and physical limitations. These models are classified by [4] as smart houses for people with low vision, movement disabilities, hearing impairments, or cognitive impairments. As stated by the authors of [4], it is difficult to make a clear distinction between these groups of disabilities because individuals with one disability often suffer from other limitations or chronic health problems. Thus, research has been undertaken to attempt to address different types of disabilities; for example, [5] proposed a framework to manage a collection of devices and applications in a home environment for people with different disabilities (blind, nonspeaking, etc.). However, this approach did not have the benefits of today’s IoT systems, which allow continuous connectivity of all devices, and thus allow uninterrupted telemetries and can enable a response in real time.

Another approach is to use so-called assisted-living systems (ALSs), which focus on providing supervision or assistance to residents to ensure their health, safety, and well-being. To accomplish this, ALSs provide services such as tracking, fall detection, and security [12]. In addition to physiological measurements, the daily physical activity of chronic patients represents an important reflection of the quality of their daily lives. Although these health measurements can help people with any disability, these approaches do not seek a complete solution, but rather aim to address a single physical or cognitive disability.

Another discipline focused on the use of technology to provide remote assistance is ambient intelligence solutions (AmI). AmI is defined as a digital environment that is context-sensitive, adaptive, and responsive to the presence of people [13], and has been used to develop cognitive stimulation technologies for the elderly. This technology is mainly focused on informing family members and caregivers about the health status of people with disabilities [3]. Unlike our proposal, this approach does not provide a personalization framework that allows health personnel to configure the system to each disability.

In recent years, several IoT proposals have been defined for health monitoring of patients with different diseases; for example, [14,15] aimed to reduce the energy cost and improve the efficiency of healthcare devices, thus reducing the need for maintenance, which is essential when dealing with patients with disabilities. However, to our knowledge, no proposals exist that propose IoT HMS frameworks dedicated specifically to people with disabilities. The iHome Health-IoT [16] framework can be applied in various health-related scenarios, including environmental monitoring, vital-sign acquisition, medication management, and healthcare services. However, its solution does not consider scenarios related to people with disabilities.

Finally, it should be noted that the survey in [17], which was used to study existing SH solutions for people with disabilities, highlighted that: (i) the main challenge of SHs for disabilities lies in the personalization that the environment can offer to adapt to the needs of the user; (ii) the SH system has to evolve according to the user; and (iii) monitoring systems for patients who depend on caregivers appear to be the more intelligent approach for the future development of SH systems. These principles were fundamental to developing our approach.

### 2.2. Necessity and Existing IoT Simulators

As stated in [17], smart devices for disabilities are expensive, and if they are not efficiently used, the system may be ineffective. IoT developers must design to achieve the maximum output while using the minimum number of sensors and actuators. For this reason, the authors of [6] propose the use of an effective simulation tool to represent complex scenarios before starting the development of IoT applications. The simulator permits the quantitative and qualitative evaluation of components in real time and ensures that the developed system meets the design requirements. A number of issues can be addressed via a simulator, including capacity planning, “what-if” simulations, analysis, proactive management, and support for many specific security-related evaluations.

One of the simulators’ possible classifications is based on the part of the IoT system architecture that they are intended to emulate. Following the division proposed by [7], a generic IoT architecture can be divided into four layers: IoT devices, gateways, communication, and backend systems.

Thus, it is notable that numerous IoT simulators focus on the communication layer, for which mature tools have been examined in previous research on wireless-sensor systems. Thus, NS-2 [18] and NS-3 [19] are simulation tools used for research purposes that permit developing a real-time network simulator. Despite their relevance, these simulators are not adapted to the specific scenarios of an HMS for people with disabilities.

Regarding the backend systems, numerous IoT simulators exist for data processing that are focused on describing sensors and edge-computing aspects. An excellent simulator is Osmobility [20], which focuses on simulating mobility, communication, and energy consumption of IoT devices. A recent work [21] proposes a dependability evaluation simulator to model fault behaviors of sensing devices and network links. Regardless of the suitability of these simulators, they cover a specific spectrum of IoT systems that does not align with our needs but could, however, complement this work.

One of the most relevant simulators in the area of healthcare is SimIoT [22]. SimIoT models the sensor-data-processing scenario relevant to remote healthcare IoT systems to evaluate its load scalability. The use case is an HMS for emergencies in which short-range and wireless communication devices are used to monitor patients’ health. Unlike our proposal, it is not adapted for people with disabilities, nor presents a friendly environment for health experts.

### 2.3. Model-Driven Engineering for Developing IoT Systems

It is well known that MDE [8] comprises a development process based on the use of models as a central element. The main principles of MDE are abstraction and automation, with an integrative development process that allows for:

- Specifying the system model, in which the heterogeneous elements are precisely identified, defining an automation process to obtain the final solution;
- The application’s complexity to be addressed;
- Facilitating communication between the application stakeholders.

In recent years, MDE has been applied to represent the complexity of IoT systems. One of the most important approaches is the IoT reference model [9], which represents a mature and well-defined reference model for IoT based on the analysis of researchers and the industry’s needs. Although this approach does not establish any automation process to obtain an IoT system, this work inspired us to define a set of formal models as inputs to the simulator of the IoT HMS for disabilities.

Most MDE approaches for IoT propose complete solutions. However, these are focused on a specific IoT domain and cannot be used directly to address the application of HMSs to individuals with disabilities. Examples of these approaches are: (i) SysML4IoT [23], which uses a new UML profile that permits validation of the quality of service (QoS) of a modeled IoT system; (ii) PervML [24], which defines a domain-specific language based on UML, to describe pervasive systems in a technology-independent manner; and (iii) MDE4IoT [25], which is an approach for modeling, validating, and generating a subset of IoT systems known as emergent configuration.

## 3. MoSIoT: A MDE Framework for IoT Healthcare-Monitoring Systems for People with Disabilities

In this paper, we propose a framework called MoSIoT, which addresses the representation of the different scenarios faced by an IoT HMS for people with disabilities. We chose an MDE approach, which generates a data-intensive simulator with the objective to inspect the IoT HMS’s complex behavior under hypotheses called scenarios. In practice, formulating a scenario enables building a hypothetical world that the medical expert can then query and navigate.

MoSIoT was applied to three of the main challenges that IoT simulators should overcome [26]: (i) modeling entity heterogeneity, (ii) customization and extensibility, and (iii) support for online decision-making.

To overcome the first challenge; i.e., modeling device heterogeneity, MoSIoT defines a formal reference model representing different entities, relationships, and behavior of an IoT HMS system.

The second challenge is related to the necessity of customization and extensibility of the IoT HMS. The introduction of new device models and new user profiles with different characteristics is required, without knowing or changing the simulator’s internal properties. To achieve this, the simulator offers a friendly user interface that permits an expert domain to instantiate the MoSIoT domain model using a Web app. Once the models are stored, a set of transformations is defined that permit MoSIoT to generate a specific scenario’s behavior.

Finally, the third challenge requires that simulators be used in online decision-making, and are able to incorporate live data and process multiples scenarios. Thus, we integrated a prediction module into the MoSIoT framework that applies an integrated set of learning attributes to the scenario model related to machine-learning algorithms. These learning attributes allow predictions to be obtained with greater precision and in real time.

### 3.1. The Software Architecture of the MoSIoT Framework

**Figure 2:**
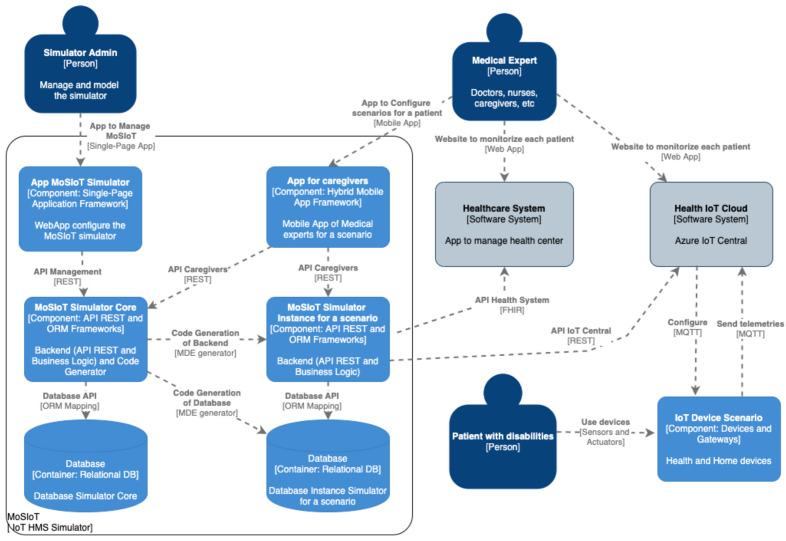
C4 Software architecture of the MoSIoT framework.

Figure 2 shows an overview of the software architecture of the MoSIoT framework using the C4 notation [27]. This architecture model represents the main components that comprise the MoSIoT framework and the actors that interact with it. Following the elements proposed by the C4 approach, we represent the MoSIoT simulator as a software container comprising a set of components. The left side of the diagram shows the core of the simulator, which comprises three components: (i) A Web app that allows an actor called the Simulator Admin to manage the simulator’s information (MoSIoT simulator frontend: https://github.com/hichman662/IoTAFA, accessed on 31 May 2021). This Web app is a progressive Web application implemented in Ionic (MoSIoT final User frontend: https://github.com/hichman662/userIoT accessed on 9 June 2021) that permits, through forms, the introduction of generic information to the simulator, thus populating the MoSIoT domain model presented in Section 3.2. (ii) The core of MoSIoT is a Web service based on a REST API interface that provides the available services to the Web app. This object-oriented business logic represents the MoSIoT domain model. (iii) A database in which all the domain information is stored and mapped through an object–relational mapping framework (e.g., NHibernate). The domain model is defined with the OOH4RIA [28] approach, which allows us to quickly update and generate its backend implementation (MoSIoT simulator backend: https://github.com/santiagomelia/MoSIoT-Backend, accessed on 31 May 2021).

The simulator offers a user-friendly hybrid mobile app (e.g., Ionic, Flutter) to the medical expert (practitioners, nurses, or caregivers) for the provision of healthcare to people with disabilities (details are provided in Section 4.1). This mobile app allows the introduction of an IoT HMS scenario for a specific type of patient based on the generic information of the domain model. For this reason, the mobile app is first connected to the simulator’s core backend to obtain all the generic information about these elements (patient profile, device templates, care plan templates) to accelerate the creation of a scenario instance. Once the health expert has created and managed the appropriate entities for an IoT HMS scenario, the execution of the MDE transformations obtains the MoSIoT simulator instance backend, which allows animation of the behavior of that particular scenario. Specifically, each MoSIoT simulator instance is a Web service comprising an API REST, transactional business logic, and a relational database for its storage. The simulator business logic contains the functionality of:

- Entity operations that the simulator has enriched with a typology to improve the generation;
- The state machine models to represent the internal states of complex entities;
- Trigger–action programming to define a set of recipes that configure the behavior of the scenario.

A prediction module integrated into the scenario model through learning attributes that gather information from machine-learning algorithms (see details in Section 4.2) completes the scenario’s behavior.

As part of the result, the simulator injects, through an API REST, all the elements and animated data involved in an IoT HMS specific scenario into an IoT Hub Cloud (e.g., Azure IoT Central). The real scenarios’ information allows us to compare their data with the predictions of our simulator, indicating what is accomplished, and allowing the medical expert to adjust and improve the simulator.

As indicated by the authors of [29], it is crucial to identify an adequate formalism to express the simulation model conceptually. In the MoSIoT simulator, this formalism is provided by the MDE paradigm. MoSIoT applies a set of models and model transformations that facilitate the transition from the medical expert’s requirements to the final IoT HMS simulator.

The following section provides a detailed description of the MoSIoT domain model, the formalization of which allows the management of the simulator’s core concepts.

### 3.2. The MoSIoT Domain Model

Following the same philosophy as the IoT reference model [9], the domain model is an essential part of any reference model. It defines the main abstract concepts (abstractions), their responsibilities, and their relationships. The domain model’s fundamental idea is to represent concepts that do not change over time, so they must be independent of both technology and specific scenarios.

In our case, the MoSIoT domain model is divided into three fundamental parts or packages:

- The patient profile package, which allows for the definition of the adaptation profiles of patients based on the types of disabilities and conditions;
- The device package, which defines the device templates or types of devices used in these systems with their characteristics and the types of telemetries;
- The healthcare package, which proposes different care plan templates with activities, goals, and communications for patients with specific conditions and disability types.

Each of the packages of the MoSIoT domain model is based on a set of industry standards that have been considered relevant in their area.

#### 3.2.1. The Patient Profile Package

**Figure 3:**
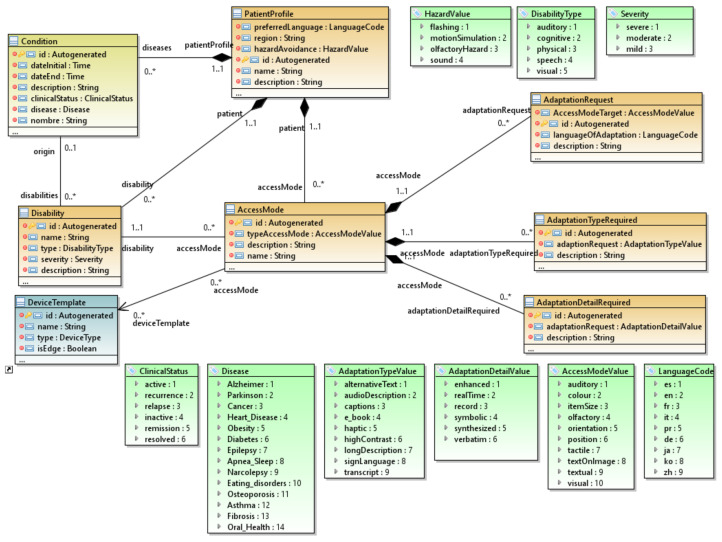
The patient profile package.

Figure 3 shows an extract of the package in which entities are expressly represented that we consider fundamental to defining the profile of a type of patient with one or more disabilities and several diseases. Although no standards to address disability for IoT currently exist, we relied on initiatives such as the ISO/IEC 24751 AccessForAll (AFA) [30] specification, which proposes a reference model that represents users’ personal accessibility needs and preferences, and controls the presentation of the information. Although the proposal was defined for the domain of e-learning resources, we extracted the domain-independent aspects from the reference model, such as the definitions of access modes and the different types of adaptations according to a specific disability.

The central element of this package is the *PatientProfile* class, which brings together the patient’s set of disabilities and the set of diseases that they may have over time. We based this element on the Web Accessibility Initiative (WAI) W3C classification [31] to define the different types of disabilities: *auditory*, *cognitive*, *physical, speech*, and *visual*. Furthermore, severity can be classified as: *severe*, *moderate*, or *mild*. The *Condition* entity is based on the Fast Healthcare Interoperability Resources (FHIR) standard [32], which indicates patients’ current clinical status (*active*, *recurrence*, *relapse*, etc.), its severity, and the disease suffered. This *Condition* entity belongs to the Health package, explained in Section 3.2.3, which defines the elements to establish a patient’s care plan.

One of the aspects defined from the AFA standard is the definition of a set of *AccessModes*, which are the accessible modes with which the patient will interact with the different devices. Each *AccessMode* will establish the relationship between the *PatientProfile* and the *DeviceTemplate*. Each *AccessMode* has an *AccessModeType*, which establishes an access mode for the patient to interact with the device. For example, a *Voice Interpreter* has an *Auditory* mode, and a smartphone may have several modes, such as *Tactile*, *Visual*, and *Auditory*. Each *AccessMode* is further composed of three elements: (i) *AdaptationRequest*, which establishes a preference of the access mode with which the patient interacts with the device. For example, the patient can indicate that if a smartphone offers a visual *AccessMode*, she chooses an *AccessModeTarget Auditory* mode. (ii) *AdaptationTypeRequired* sets a form of adaptation determined by the disability; e.g., a smartwatch may have a visual *AccessMode*. A person with a visual impairment may set an *AdaptationType* equal to *AudioDescription*. (iii) *AdaptationTypeDetail* is a more specific adaptation of the previous *AdaptationType*. Following the previous example, we can set the *AudioDescription* to have an *AdaptationDetailValue* of type *Record* so that all descriptions are recorded and can be subsequently heard again.

Therefore, each patient profile of a specific clinical picture with disabilities establishes an accessibility configuration with a set of device templates. Thus, when a healthcare professional establishes a specific patient’s profile, they will be able to choose the accessibility configuration that best suits that patient a priori. This makes it possible to obtain previously defined expert knowledge and substantially reduce the effort required to define the patient in the scenario model.

#### 3.2.2. The Device Package

**Figure 4:**
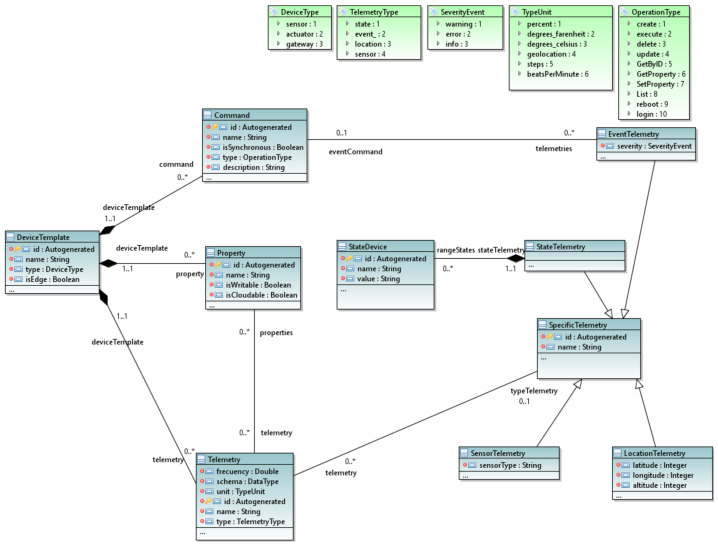
The device package.

The device package consists of the entities necessary to represent the IoT devices that constitute the MoSIoT simulator. Figure 4 shows an extract of this package, the central element of which is the *DeviceTemplate*. Its definition is based on the W3C Web of Things standard [33], but adapted to commercial solutions such as Azure IoT Central [10], the platform used for the current implementation of MoSIoT.

Specifically, a *DeviceTemplate* is an abstraction that defines all the characteristics and behaviors that a device type needs within an IoT system. The *DeviceTemplate* provides a set of *Commands* that are the methods of the device that are offered to the rest of the system to represent the behavior. For each *Command*, we define whether it is asynchronous. It is assigned a type (e.g., *create*, *getter*, *delete*, *reboot*, etc.) that endows it with a specific behavior (e.g., a reboot command puts the device in its initial state). The *DeviceTemplate* contains a set of *Property* elements, which are the different internal data fields that define the device’s state (e.g., a smart light bulb has the property state with the values on and off).

Furthermore, each *DeviceTemplate* contains a set of *Telemetry* entities, representing a human-readable view of the device information relevant to the client. In this context, these are typically significant patient measures (heart rate, steps, blood pressure, etc.) or metrics of the device (battery, temperature, internal state, etc.) represented in the scenario control panel. Thus, each *Telemetry* must define the properties it represents, the type of data (schema), the frequency of collecting data by the device, and the data unit.

Different *Telemetry* subtypes can be specified through the *SpecificTelemetry* entity. Four types are defined: (i) *StateTelemetry* allows the definition of the range of *StateDevice* to be displayed by sending the IoT Hub the states that the device goes through. (ii) *EventTelemetry* establishes a trigger sent to the Hub when one or more commands are executed on the device. (iii) *LocationTelemetry* is necessary for devices that need to store their location. (iv) *SensorTelemetry* establishes *Telemetry* to collect information from a specific type of sensor; for example, we define the *SensorType* equal to temperature for a thermostat device.

#### 3.2.3. The Healthcare Package

The third package of the MoSIoT simulator defines the entities that enable patient care and the establishment of specific care plans to be monitored by the IoT system.

For its definition, we analyzed several standards that allow the storage of patients’ medical information, such as ISO13606 [34], OpenEHR [35], and CDA of HL7 [36], which are based on the definition of complex reference models based on archetypes with a stronger focus on their persistence. These standards provide a sizeable expressive capacity to specify patients’ clinical data, but the interoperability of their data is considered secondary.

In contrast, in the context of IoT HMS, interoperability is considered fundamental because devices send data to IoT hubs and the healthcare systems to which patients belong. Therefore, based on a current health standard; i.e., HL7 Fast Healthcare Interoperability Resources (FHIR) [32], the aim is to simplify and accelerate HL7 implementation using open Web standards such as REST, JSON, HTTP, and OAuth.

As the name implies, FHIR is focused on interoperability, simplifying communication with the rest of the simulator and, in addition, providing the possibility of connecting with healthcare systems, both to retrieve information from patients and healthcare professionals, and to send information if necessary.

Specifically, among the set of modules existing within the FHIR standard, we focused on the Workflow module, which is responsible for the coordination of activities between the different systems. We focused on defining a care plan that allows the definition of a set of clinical or administrative activities to manage patient care, followed by monitoring of the implementation of these plans. Following the FHIR HL7 standard, most entities have included an encoding based on standards such as SNOMED CT [37] for clinical terminology, ICD-10 [38] for diseases, and LOINC for care plans [39], thus providing a comprehensive and precise clinical health terminology.

**Figure 5:**
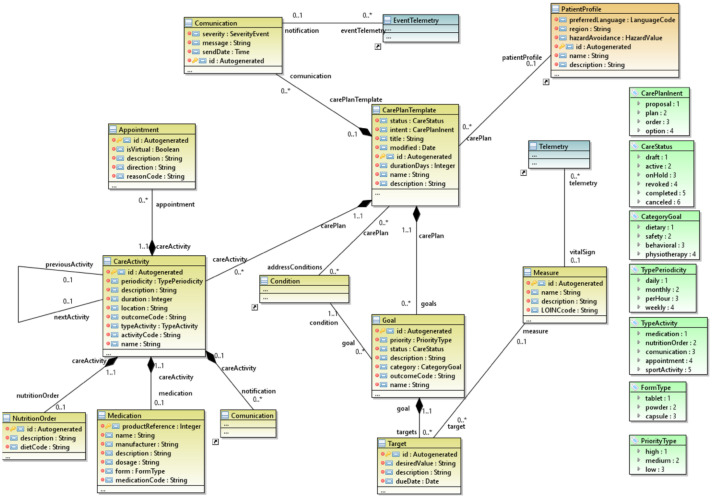
The healthcare package.

As shown in Figure 5, the health package’s core element is the *CarePlanTemplate,* which is based on the FHIR CarePlan to define all the elements that will enable the establishment of an IoT-assisted care plan. The *CarePlanTemplate* also defines a generic treatment plan for specific disabilities and critical illnesses. Once defined, the MoSIoT scenario will instantiate for a specific scenario whose specific needs will be defined.

The *CarePlanTemplate* defines the duration (in days) of the treatment, the status of the plan, the list of *Conditions* or diseases it intends to address, its intent, and the modification date. It is important to note that the domain model relates a *CarePlanTemplate* to a given *PatientProfile.* This allows a filter to be established when we select a care plan, following the establishment of a patient profile in the definition of a scenario.

The CarePlanTemplate is a composite element containing three lists of entities: (i) a list of *Activities* called *CareActivity*, which are the actions to be performed as part of the plan; (ii) a list of *Goals*, which are the objectives to be met in carrying out the plan; and (iii) a list of *Communications* that permit messages sent to other users to be registered.

*CareActivity* represents the action by the patient over a certain period, duration, and periodicity. Both *activityCode* and *outcomeCode* codification follow the SNOMED CT standard. *CareActivity* can contain extra information on other entities such as: (i) Medication—a prescribed medication that the patient takes during a specific period; (ii) *NutritionOrder*—the care plan instructs the patient to perform a specific food diet; and (iii) Appointment—a proposed virtual appointment with a practitioner.

As mentioned above, a *CarePlanTemplate* contains a set of *Goals*. Each *Goal* defines a priority, a category (*dietary*, safety, behavioral, or *physiotherapy*), its description (e.g., the patient must lose weight), and outcome, which is also coded by SNOMED CT. Each *Goal* contains a set of *Target* objects that allow the *Goal* to be set quantitatively; e.g., for a weight-loss goal, the *desireValue* indicates the number of kilograms to be lost and the date. Moreover, the *Goal* establishes a relationship with the *Measure* entity, which indicates the measurable concept to be treated (i.e., weight, % body fat, heart rate, etc.). This *Measure* entity is in turn related to a *Telemetry* that is sent to the IoT hub.

The MoSIoT domain model generates a common understanding of the target domain, collecting all the valuable information in an IoT HMS for people with disabilities. As indicated [4] in the definition of the domain model, a set of concepts exists that remains constant, regardless of the specific scenarios represented. Other concepts will vary for each case. The Web app that manages the Simulator Admin permits the introduction of all the information that remains constant (see Figure 2). For example, the different *DeviceTemplates* or types of devices used in an IoT HMS (e.g., smartwatch, smartphone, voice assistance, or any wearables), in addition to their properties, telemetry, and commands, will be the same, regardless of the scenario.

**Figure 6:**
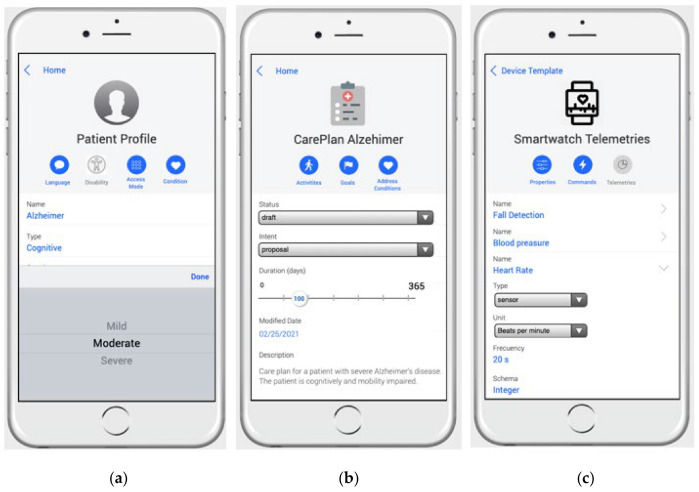
Web app admin of MoSIoT. (**a**): Patient Profile edition screen; (**b**): CarePlan Alzheimer edition screen; (**c**): Smartwatch Telemetries edition screen.

Figure 6 shows several views of the mobile version of the MoSIoT framework Web app. In Figure 6a, a *PatientProfile* is introduced, showing the parameters of the patient’s disability; in this case, Alzheimer’s with moderate cognitive impairment. Figure 6b shows a screen of a *CarePlanTemplate* for Alzheimer’s disease depicting its parameters, such as indicating that the plan status is draft, its intent is a proposal, and the duration is 100 days. The description indicates that this plan is for patients with severe Alzheimer’s disease with cognitive and mobility impairment. Finally, Figure 6c shows the screen of a smartwatch device in which its telemetries, specifically the telemetries *Fall Detection, Blood pressure*, and *HeartRate*, are defined. The screen shows the details of the *HeartRate*. This is a type of sensor telemetry; the unit of the metric is beats per minute; the frequency with which it collects information is 20 s; and the schema that defines its data type is Integer.

Once the domain model has been defined, the next step is to represent the scenarios for each patient. For this purpose, the following section defines the metamodel of the scenario model, which gathers the knowledge of the domain model and instantiates the concepts specific to each scenario.

### 3.3. The MoSIoT Scenario Metamodel

As stated in the IoT reference architecture [9], the information model is a model that defines the main elements of information needed to define an IoT system scenario. MoSIoT has renamed the information model as a scenario model, which is the instantiation of the concepts that vary in each of the IoT HMS scenarios. For the construction of the scenario model, our framework establishes a relationship between the scenario entities and the domain entities. This aspect provides several advantages, such as: (i) speeding up the scenario model’s creation, because many concepts are automatically generated from the domain model information; (ii) simplifying the definition of scenarios by offering the practitioner a choice between possible alternatives for patient profiles, care plans, and device templates; and (iii) the scenario model feeds back to the knowledge base of the domain model with the data obtained from the execution of the different real cases.

The first step in defining the scenario model is to define its abstract syntax; i.e., its metamodel. This metamodel is split into two fundamental parts: the core, in which the main elements that will structure the dynamic and static part of the model are defined; and the relationships between the scenario model and the domain model, in which the invariant domain information is provided. In this case, these are the elements of an IoT HMS, and the restrictions obtained by the scenario’s fundamental entities through this relationship.

**Figure 7:**
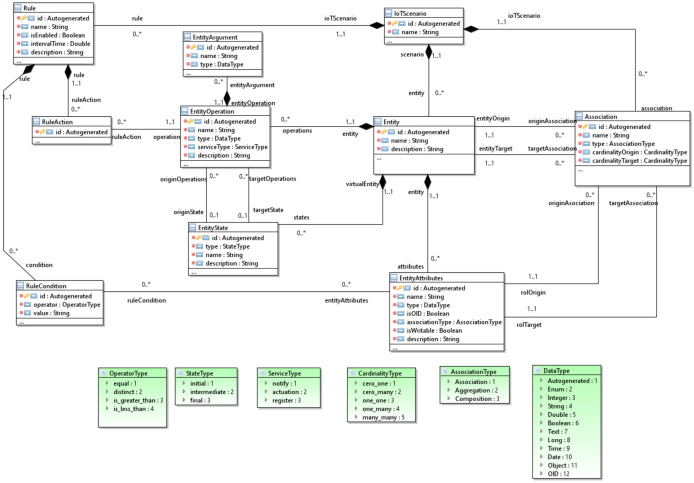
The metamodel core of the MoSIoT scenario model.

Figure 7 shows an extract of the core of the scenario model metamodel. Its central element is the *IoTScenario* class, which contains all the elements that are part of a scenario. The *IoTScenario* contains a set of *Entity* objects, each of which defines a real-world entity that is part of the IoT scenario. The *IoTScenario* has a set of *Association* objects that represent the relationships between the different entities.

Each *Entity* of the scenario model has a static part, defined by a set of *EntityAttributes* that can be primitive data or refer to other *Entities* through the *Association*. An *EntityOperation* represents the dynamic part of the *Entity*. These operations also contain a set of *EntityParameters*. In addition, the *Entity* has a setting of *EntityState*, representing the different states that an entity can go through during its life cycle. The different *EntityOperation* values trigger the transitions of these states at both the source and destination of each state.

The core contains the IoT system’s behavior, represented by the set of rules called recipes. We relied on trigger–action programming for the definition of the recipes [40]. This programming paradigm is based on frameworks such as IFTTT, Integromat, and Zapier. This paradigm allows medical experts with no programming knowledge to introduce new recipes that allow the proper configuration of each IoT HMS. A *Recipe* is a combination of a *Trigger* and an *Action* that follows the format IF (TriggerFunction) Then (ActionFunction). A *Trigger* is either an event or a condition. The event relates to an *EntityOperation*, and the condition is defined by an *operatorType* (equal, distinct, isGreaterThan, isLessThan, etc.) from the value of an *EntityAttribute*. A *RecipeAction* is a command or operation that will be executed when the trigger is fulfilled. This operation belongs to an *Entity* of the IoT system. This core allows the establishment of a static and dynamic representation of the concepts to be represented in a scenario; however, the *Entity* subtypes that are finally instantiated in the IoT HMS scenario must be established.

**Figure 8:**
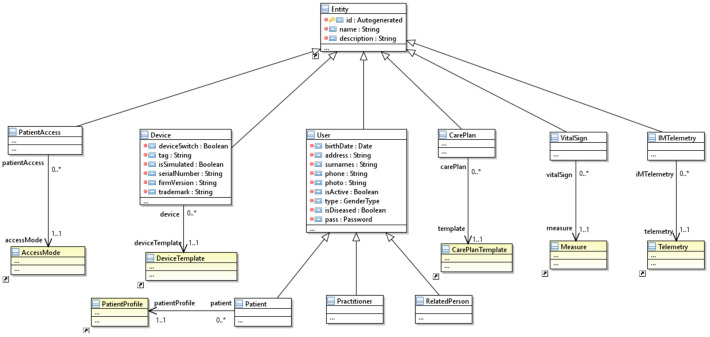
The entity hierarchy metamodel of the MoSIoT scenario model.

Figure 8 shows the metamodel’s extract of the scenario model in which the specific entity types of the IoT HMS domain are defined. Each of the entity types of the scenario model is related to an entity of the domain model. The simulator obtains the previous domain information from which its entities are generated, and the possible relationships between the different entities are established. Thus, a patient entity has a relationship with the *PatientProfile* entity of the domain model. Due to this relationship, patient retrieves the information of a specific *PatientProfile*, such as the type of disability with its access modes, and the disease or condition. In this way, when the physician defines a *PatientAccess*, which has a relationship with *AccessMode*, the model will propose a set of *AccessMode* entities within the previously chosen *PatientProfile*. The medical expert can reuse one of these or create one from scratch.

The scenario model can define a set of *Device* entities representing the Thing of WoT or VirtualDevice of the Reference IoT Model. Each *Device* has a unique identifier within the IoT hub and the entire Internet. In addition, it has the data of the physical device, such as the *serialNumber* and the *firmVersion*. However, it can also be a simulated or use a real device, with the attribute *isSimulated* equal to false. Moreover, each device is related to a *DeviceTemplate*, which gathers information about the commands, properties, and telemetries. Thus, when a device with its *DeviceTemplate* is chosen, a medical expert can define an *IMTelemetry*, which they can choose from the subset of *Telemetry* contained in that *DeviceTemplate*.

Finally, the third domain in which the simulator can restrict elements is contained in the healthcare package. Specifically, when a *CarePlan* is created, it can be assigned to a *CarePlanTemplate* related to the disabilities and conditions of the patient. Once this *CarePlanTemplate* is chosen, then *CarePlan* elements such as *Goals*, *Activities*, and *Communications* will be proposed to the medical expert, or new elements can be created.

Once the semantics of the scenario metamodel have been defined, the scenario model can be instanced and applied to a real case. The following section explains a scenario model representing an IoT HMS of a patient with Alzheimer’s disease.

## 4. A Case Study of the MoSIoT Scenario Model: A Patient with Alzheimer’s Disease

Alzheimer’s disease (AD) affects millions of people globally and is currently the sixth-leading cause of death in the USA, having increased by 98% since 2000. For this reason, it is necessary to implement measures that not only help to combat the disease, but also help improve the quality of life of those affected, by allowing practitioners to monitor their progress on an ongoing basis.

For the definition of this scenario, we relied on the work of Kourtis et al. [11]. These authors have conducted research in the medical field; therefore, all treatments and follow-ups indicated by IoT HMS were based on scientific evidence from real patients. The work focused on studying the evolution of patients with AD who suffer a gradual reduction in their cognitive and motor skills.

### 4.1. The MoSIoT Scenario Model

The MoSIoT scenario model allowed the representation of a scenario in which a specific care plan for AD was specified. This scenario was associated with the capture of a set of telemetries collected by one or more devices that specifically related to this disease or condition. In addition, as one of the main contributions, accessibility scenarios were considered, which in this case could be both physical and cognitive, so that the patient could receive information and communicate with other people.

The model allowed the capture of variabilities that occurred between patients with AD; i.e., the model could be defined by a doctor for several patients with the same disease stage. However, the model was instantiated with each patient and could evolve as the patient’s disease progressed, thus addressing intrapatient variability.

**Figure 9:**
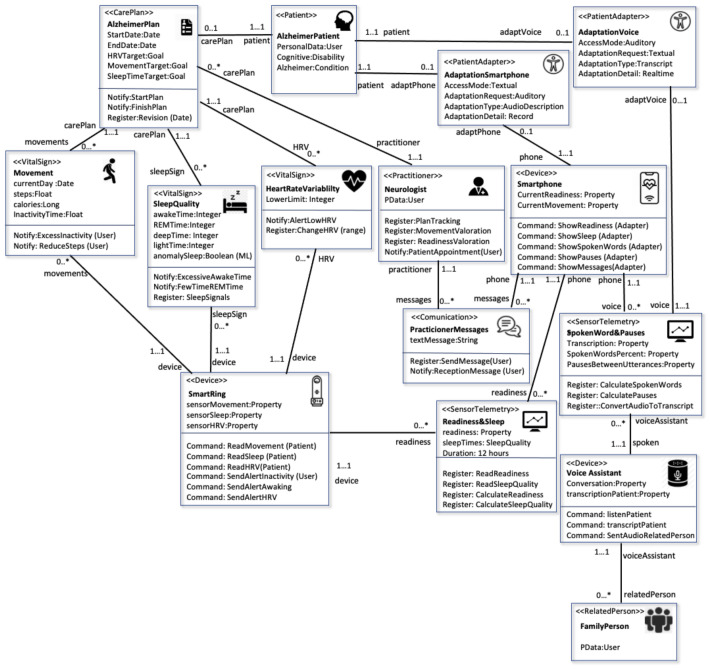
Alzheimer’s scenario using the information model.

The current scenario (depicted in Figure 9) started with the main element, which was the *Patient*. When this was defined, a patient profile had to be selected from those available in the domain model; at that moment, we chose the profile for AD. Subsequently, the simulator offered a subset of the access modes available for that type of patient, although a doctor could modify an access mode that they consider appropriate.

Associated with this patient, we have the element that provides input to the patient’s follow-up configuration, in this case, the *CarePlan*. As previously indicated, the *CarePlan* can be defined from *CarePlanTemplate* defined in the domain model, which provides a possible configuration for this disease. The *CarePlan* contains a period of validity between the start date and end date, and a set of *Goals* associated with different *VitalSign* values defined for this care plan. Based on a previous study [11], we established the *VitalSign* values of interest to be collected from the patient. The first was Sleep Time. Studies of AD populations have confirmed that patients with AD experience more night-time awakenings, less time in REM sleep, and lower sleep efficiency. Thus, [41] recommends using a *Smartring*, which has shown higher levels of sleep-staging accuracy than other devices located on the wrist, such as smartbands and smartwatches. In the *VitalSign* entity called *SleepQuality*, we captured metrics that corresponded to the different sleep stages, such as *awakeTime*, *REMTime*, *deepTime*, and *lightTime*. In addition, we defined a learned Attribute called *anomalySleep*, associated with a machine-learning algorithm (explained in Section 4.2). We also established notification operations in the case of an excessive number of *awakeTime* and a reduced *REMTime*. This *VitalSign* is related to the *Smartring* device in charge of collecting such information.

The second *VitalSign* defined was heart-rate variability (HRV), a measure of time intervals between heartbeats, resulting from the heart’s dual modulation by the sympathetic and parasympathetic systems. In adults, HRV is correlated with cognitive functions, attention, memory, stress, and social cognition [42]. Thus, HRV serves as a compelling marker for AD progression. The *VitalSign* HRV was included in the model, and the lower limit at which values were considered to represent negative cognitive function was defined. This HRV measure was also linked to the *Smartring* device, which was equipped with photoplethysmography and offered high reliability [11]. Another measure collected directly with the *CarePlan* was Movement. Studies based on actigraphy data using accelerometers have been useful in establishing an objective measure of apathy in patients with impaired activity levels and neural impairment [43]. Thus, the scenario defined that the *Smartring* collected data relating to steps taken during the day, calories consumed, and inactivity time. In addition, we defined a notification when the level of steps was lower than this value.

The three metrics were finally grouped into a single *SensorTelemetry* called Readiness & Sleep. The *Smartring* device monitored the body’s signals, registered daily habits, and provided an overall measure of the patient’s recovery called *Readiness*. The *Readiness* measure allowed signaling of the capacity to perform mental, emotional, and physical activities. The *Smartring* device also gathered different types of sleep (deep, light, REM) and heart-rate insights, which allowed an overall sleep-quality reference to be derived.

One of the essential entities in the scenario was the *Practitioner;* in this case, a *Neurologist* in charge of monitoring the disease, following up the *CarePlan*, and introducing assessments of the patient’s evolution in both *Readiness* and *SleepQuality*. Doctor–patient communication was introduced into the domain, and was necessary in most scenarios. For this purpose, the scenario had a *Communication* entity that sent messages between the two actors, and each actor was notified of their receipt. As previously indicated, for communication with an Alzheimer’s patient who has a cognitive disability, the patient’s communication must be accessible. Thus, we defined a *PatientAdapter* entity between the *Patient* and the smartwatch device in charge of collecting and sending messages. The *PatientAdapter* entity had defined elements from the accessibility package of the domain model. In this communication, the default *AccessMode* used by the smartphone for the messages was textual. However, due to this patient’s cognitive disability, we provided an *AdaptationRequest* equal to *Auditory* so that these messages were translated from text to speech for this patient. Finally, we introduced an *AdaptationDetail* with a value equal to Record, which indicated that the system would record speech messages for later playback.

The last part of this scenario was analyzing the Alzheimer patient’s speech and language in their conversations. According to a metric study [44], the proportion of words spoken in a patient’s conversation can correlate with transitions from normal cognition to mild cognitive impairment. A simple metric quantifying pauses between utterances shows memory problems associated with AD. In our scenario, we staged the capture of both metrics using a voice assistant that picked up the patient’s spoken words in a conversation with *RelatedPerson;* in this case, with a family member. The IoT HMS transcribed these conversations into text and stored them in a *SensorTelemetry* called *SpokenWords&Pauses*, calculating the percentage of words spoken by the patient and pauses between utterances.

We also introduced an accessibility mechanism through a *PatientAdapter* to improve the patient’s interaction with the voice assistant. The mechanism displayed the text with the spoken messages on a screen that accompanied the voice assistant, thus setting the *AdaptationRequest* as Textual and indicating that the *AdaptationType* was equal to *Transcript*. The *AdaptationTypeDetail* was equal to *RealTime*, thus establishing the speech-to-text transcription in real time.

**Figure 10:**
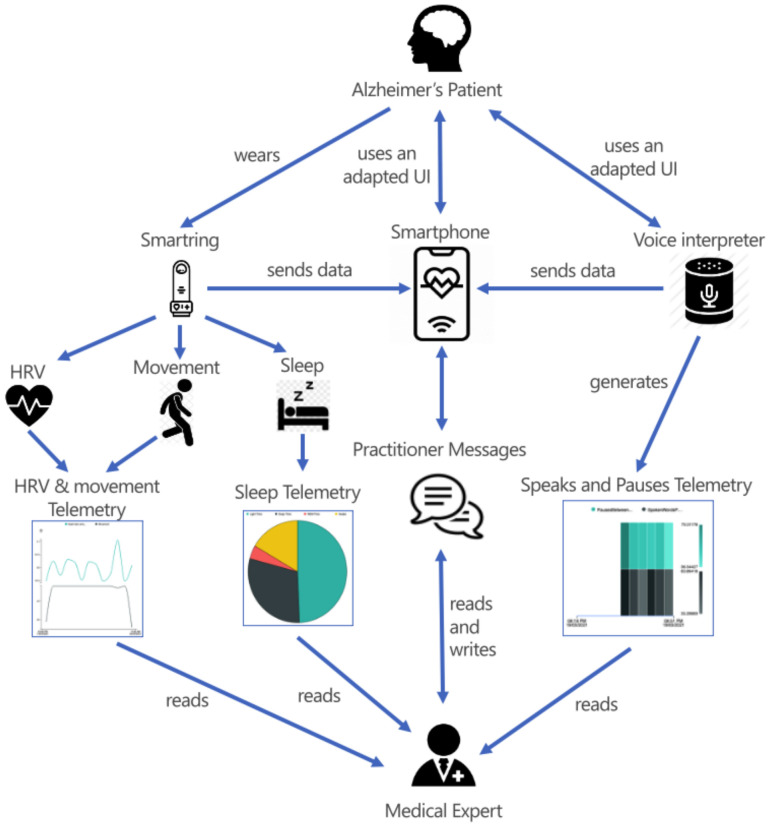
Data flow diagram of the Alzheimer’s scenario.

Figure 10 provides a schematic of the data flow between the different actors in the Alzheimer’s scenario. The diagram shows how the patient sent data from the wearable *Smartring* and two devices with an accessible interface, such as the voice assistant and the smartphone. The three devices were responsible for generating the HRV and movement, Sleep and Speak, and Pause telemetries, which the medical expert read. In addition, the diagram shows the data flow in the communication between the doctor and patient through messages.

**Figure 11:**
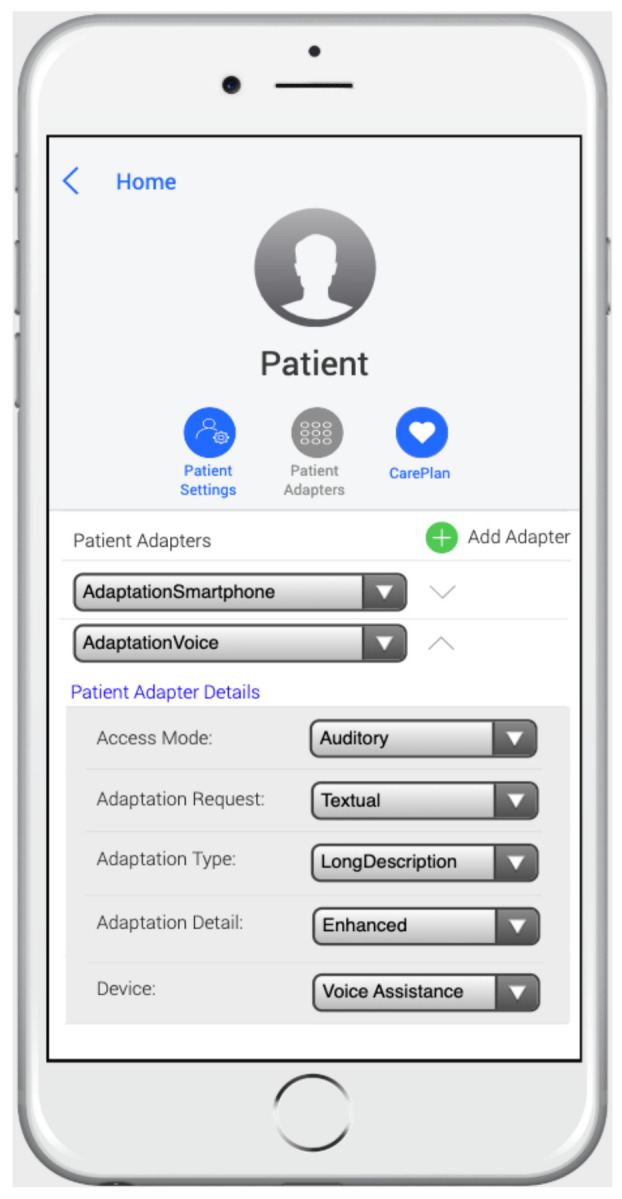
Web app for the MoSIoT scenario model.

As indicated in the MoSIoT framework architecture shown in Figure 2, the healthcare expert entered the patient scenario information using a mobile app that allowed them to quickly enter the entities and their relationships of the IoT HMS scenario. Figure 11 shows an example of a doctor’s mobile app screen in which they inserted the patient’s *PatientAdapters*. In this case, the doctor could create a new *PatientAdapter* or choose the different *AccessMode* associated with the *PatientProfile* of the patient through a Select control. Following the same Alzheimer’s scenario model as in this section, when the *AdaptationVoice* patient adapter was selected, its detail with the *AccessMode* and the different adaptations were explained. Finally, the app permitted the association of the *PatientAdapter* to a *Device* (e.g., Voice Assistance) defined in the scenario.

**Figure 12:**
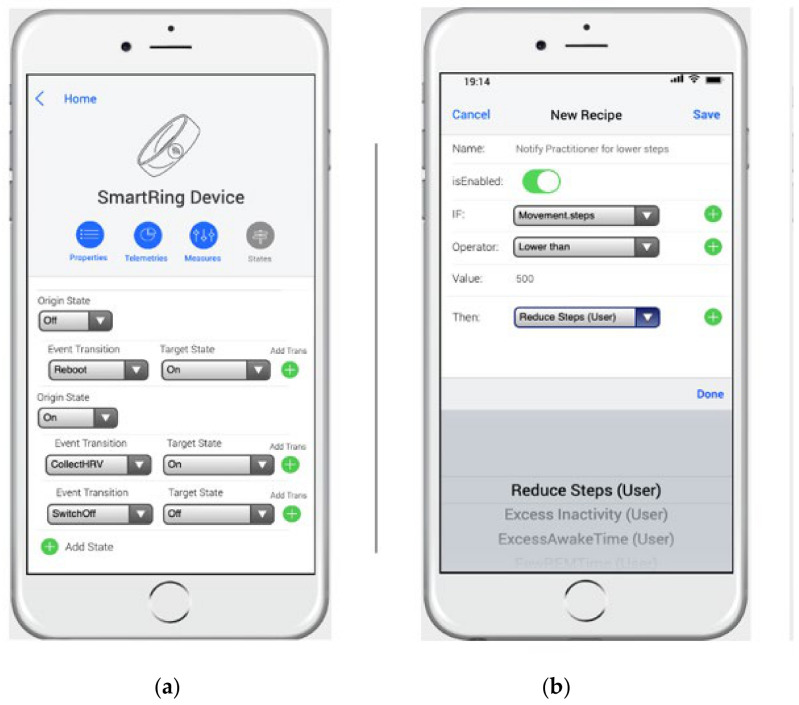
Web app for the MoSIoT scenario model. (**a**): Smartring state machine edition screen; (**b**): New recipe creation screen.

However, to complete the simulator, each entities’ internal behavior and the programming of the rules that allowed the particular conditions to be established were required. Thus, the doctor could establish a state machine for complex entities, and establish a behavior based on a set of states that respond differently to each of the operations. Figure 12a shows how the app allowed the *Smartring* state machine definition, which indicated Off as the initial state. Its transition to an On state occurred by executing a Reboot event, which could, in turn, receive two events: a ReadHRV event to collect the data from the VitalSign HRV, and a SwitchOff event to switch the device back to an Off state. The *Smartring* can collect many events, but we only show these states due to the mobile interface’s limitations.

Another aspect that defines the behavior of the simulator is the introduction of the recipes based on trigger–action programming, which, as indicated in the previous section, were introduced in the scenario model as a set of “IF(Trigger) Then(Action)” recipes. Figure 12b shows the introduction of a recipe with the name “Notify Practitioner for lower steps”. This stated that if the number of steps the user performed in a day was less than 500, it triggered a notification to the Practitioner called Reduce Steps, which was selected from the different notifications of the *Smartring* device.

Once the healthcare expert defined the scenario model, our framework initiated a code-generation process employing model-to-text transformations of OOH4RIA [28] to obtain the MoSIoT simulator instance for a scenario (see Figure 2). This component was a Web service made up of an API REST, the business logic with the scenario’s functionality, and a persistence layer based on an object–relational mapping that accessed a relational database in which all the information related to the patients in the scenario was stored. Because the scenario model defined a typology of entities (Patient, CarePlan, Device, etc.) linked to the MoSIoT domain model, detailed information was available to generate functional code for each entity. The framework generated the static part represented by the entities’ attributes, which could have primitive types or types from association relations, or those defined in the domain model. The framework also generated the dynamic part through the typed operations (Notify, Register, etc.), the state machines of those entities with a complete behavior, and the programming of the set of recipes appropriate to that scenario. Following generation, the scenario entities in an IoT hub were created, and data were provided to animate the simulation.

The following section addresses the task of generating the data for the IoT hub from the prediction module.

### 4.2. The Prediction Module of MoSIoT Simulator

The prediction module of the MoSIoT framework had inputs from three sources of data related to the system behavior: (i) the input from expert knowledge that is defined both in the domain model and by the specific entities in the scenario model; (ii) the application of prediction techniques based on machine learning (ML); and (iii) the input of data from real patients interacting with the already-implemented system.

The prediction module was integrated into the business logic generated from the specific scenario. As stated in a previous study [29], performing a proper simulation requires an accurate representation of the scenario. In our case, the MoSIoT scenario model accurately represented all entities’ behavior in an IoT HMS scenario. However, it was necessary to adopt an approach that first provided the past data, and then animated that data through the events expressed by the recipes and the different entities’ internal behaviors.

For this purpose, because MoSIoT is an MDE approach, we applied an approach based on [45], which seamlessly integrated machine-learning algorithms in a domain model; specifically, in data sets composed of independent and heterogeneous entities with very different behaviors, as was the case of the IoT HMS. The traditional coarse-grained machine learning that searches for commonalities across the whole dataset is often imprecise and rigid, and requires the process to be recomputed each time a single element of the domain model is changed.

However, this approach [45] follows fine-grained learning based on a “divide and conquer” technique, which is capable of performing complex learning tasks with a reusable, chainable, and independent set of microlearning units integrated in different entities of the scenario. This technique establishes the mapping between the specific machine-learning algorithm and one or several domain elements, allows it to execute the microlearning units fast enough, and can apply live or online learning.

**Figure 13:**
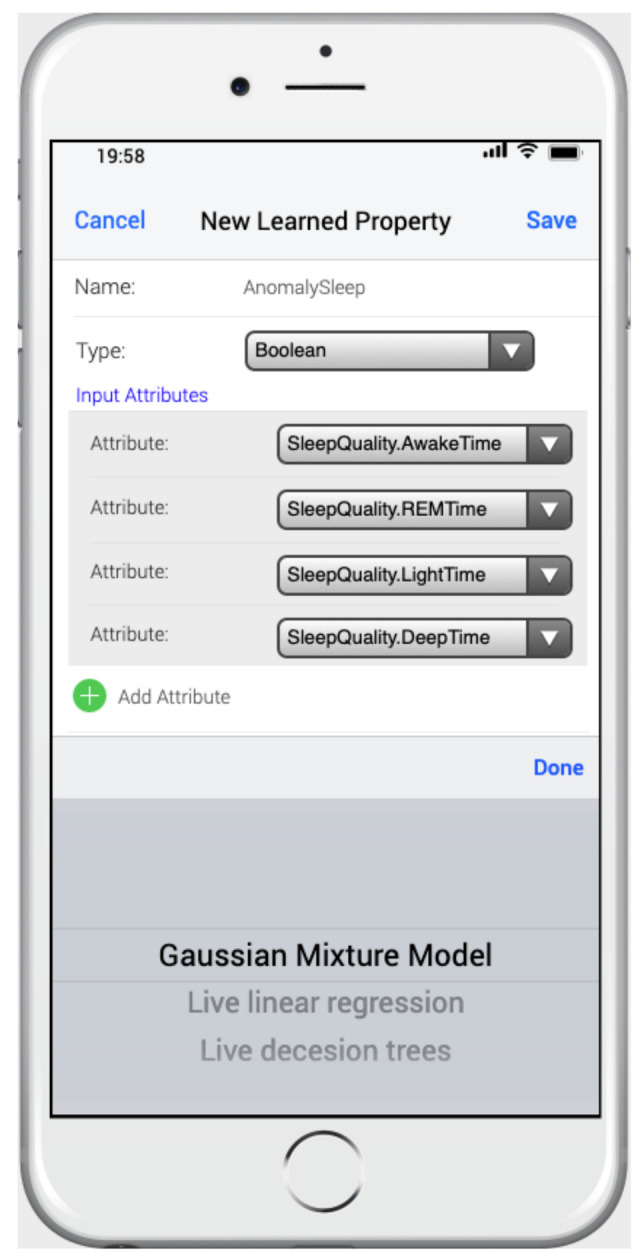
Web app for the MoSIoT scenario model.

In our case, the model used to integrate the microlearning units was the scenario model, for which an extension was defined in its metamodel, relating to the information to be learned (learned attributes), the manner in which it should be learned (ML algorithms), and what was to be defined (attributes). Specifically, Figure 13 shows an example based on the case study presented in the previous section. A learned Attribute called AnomalySleep of type Boolean was defined, which allowed the detection of anomalies in the sleep of Alzheimer’s patients. For this purpose, we established a set of input attributes that related to the different sleep states of a person, which were obtained by the measure SleepQuality (AwakeTime, REMTime, LightTime, and DeepTime). In addition, a machine-learning algorithm called the Gaussian mixture model, which allowed the detection of anomalies using an unsupervised clustering approach, was applied. The algorithm stored the AnomalySleep attribute’s information, setting its attribute to true when an anomaly occurred in the patient’s Sleep Quality.

It is important to note that the framework used the learned Attributes within a scenario model as another attribute. Thus, this could be integrated into the IoT HMS to send a notification to the practitioners with the NotifyAnomaly event when the value of the learned attribute AnomalySleep was equal to true; i.e., an anomaly had occurred in the patient’s sleep. Transitions could also be made between the states of the entities or sampled in the telemetries.

### 4.3. The IoT Hub Integration

Once the scenario’s business logic configured its dynamic part and its prediction module, the next step was to provide data to the IoT hub. Specifically, in the current implementation of the MoSIoT framework, Azure IoT Central was chosen due to its suitability to the medical environment and its compatibility with the FHIR standard. The business logic component in .NET, using an extension of OOH4RIA that integrated the aspects of machine learning, was responsible for providing the data contained in its database via invocations made from its business logic to the API REST provided by Azure IoT Central (see Figure 2).

**Figure 14:**
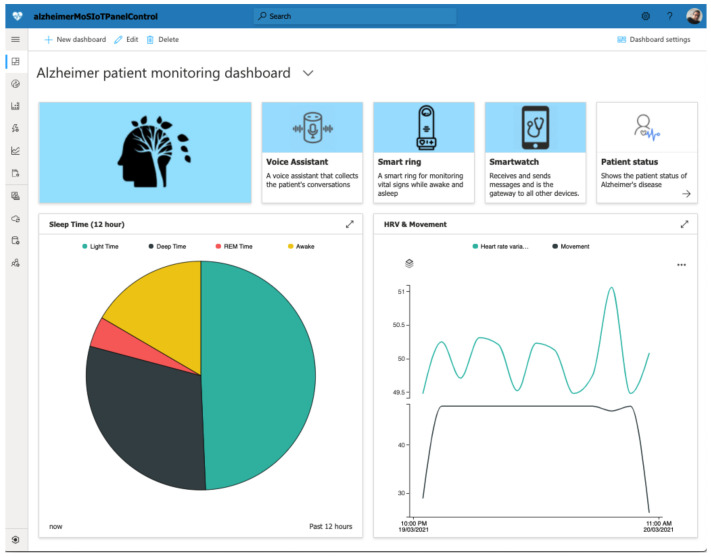
An excerpt of the dashboard for the Alzheimer’s patient.

Following the Alzheimer’s patient case study, Figure 14 depicts an excerpt of the practitioner’s dashboard with the patient’s telemetry information. As previously indicated, MoSIoT customized the dashboard, showing the scenario model telemetries proposed for this scenario. In this case, three devices sent information: the Smartring, the Voice Assistant, and the Smartwatch. This dashboard screenshot shows two telemetries. A telemetry with the types of Sleep phases is represented in a pie chart. Specifically, this showed that, during the previous 12 h, the patient had almost 49% of Light Sleep hours, 27% of Deep Sleep, 17% of Awake Time, and only 5% of REM Time. The panel also shows another telemetry represented by a line chart, which compared the evolution during 1 h of the patient’s movement, represented by the Movement vital sign and the evolution of their HRV. Specifically, it shows that the patient started the hour by performing a constant movement derived from an activity such as a walk, while their HRV was similar, with the exception of the last phase, in which the difference between the pulsations increased, coinciding with the end of the activity.

The IoT hub integration with the MoSIoT framework provides two benefits. First, MoSIoT provides the practitioner with a control panel for each patient on a website, provided in this case by Azure IoT Central, which allows them to see the evolution of each patient’s telemetry in real-time. In addition, the cloud is able to communicate with the real devices through the MQTT protocol, or other alternative protocols compatible with the devices and the IoT cloud. The simulator implements a real scenario in which patients with disabilities can use these IoT scenarios to provide real data to the simulator.

These aspects provide a scalable solution and allow multiple patients to benefit from an IoT HMS solution. However, one question remains to be addressed: the connectivity of the different patient devices to the IoT Hub to provide tracking data. In the current implementation, we relied on the scalable Azure IoT Central solution, which supported an automatic registration in which the device was registered automatically when it first connected. This scenario enables an original equipment manufacturer (OEM) to mass-manufacture devices that can connect without being registered. An OEM generates suitable device credentials and configures the devices in the factory. Optionally, it can require an operator to approve the device before it starts sending data. Specifically, our solution configured a shared access signature (SAS) group enrollment for each group of patients that belonged to the same MoSIoT scenario.

## 5. Discussion

Following the above presentation of the proposal, we now will indicate the main challenges to validation that need to be evaluated.

First, we decided to evaluate our approach using an end-to-end real case study of an Alzheimer’s patient with a cognitive disability. Nonetheless, we showed the usefulness of the approach and relied on recognized medical studies to demonstrate the solution’s usefulness. One remaining challenge to validation is that the evaluation case study might be especially appropriate for the presented solution. Thus, we need to consider additional case studies to better estimate the presented approach’s general applicability.

Another aspect that may challenge external validation is that MoSIoT’s implementation only supports one of the most recognized IoT platforms; that is, Azure IoT Central. However, this aspect could limit the solution in some areas. The Azure IoT platform has not validated all commercially available devices for different disabilities and diseases. Therefore, it is important to note that we implemented the MoSIoT framework as a set of independent Web services, allowing it to connect to any IoT hub, such as AWS IoT and Google Cloud IoT. However, adapting to another IoT hub would require implementation of new transformations in the simulator generator to send data to the new hub.

The main difference between previous related IoT HMS proposals, such as [14,15,16], and our approach, is that the former presented ad hoc solutions that achieved highly optimized results for particular devices, and thus required significant manual development to introduce new devices. In contrast, MoSIoT delegates the development of such devices to third parties, which implies a possible loss of optimization, but focuses more on the ability of the IoT HMS to be flexible to changes in the scenario. Due to MDE, MoSIoT can change the scenario model, generate modified code, and quickly adapt to each patient’s specific needs.

## 6. Conclusions

This paper presented the MoSIoT framework, which allows the representation of different IoT HMS scenarios for people with disabilities. MoSIoT focuses on the application of an MDE approach that provides the following: (i) A formalization of the different domain elements that constitute the context of healthcare-monitoring systems for people with disabilities. A domain model was proposed as the core of the application, which, based on the standards, defines the contexts of accessibility, the IoT devices, and the healthcare domain. (ii) A scenario model that allows the static and dynamic definition of all the entities to represent a patient with a specific type of disability and disease. In this study, we applied the model to an Alzheimer’s patient with a cognitive disability. (iii) The integration of machine learning in the scenario model allowed the integration of ML algorithms directly in the entities. It was thus able to obtain more accurate and faster predictions based on data obtained in real time. Finally, the framework was implemented based on the extension of the OOH4RIA approach, which allowed generation and management of the models as if they were business applications, with a scalable backend accessible through an API REST, a transactional business logic, and a relational database for their persistence. In addition, it is possible to manage the models through hybrid mobile applications that allow users without modeling knowledge and experts in the health domain to easily create a scenario. Finally, through the prediction module, the framework animates a scenario, sending the data to more powerful IoT hubs, such as Azure IoT Central, which allows the information of each patient to be displayed in the web console. Moreover, this permits fast integration of multiple devices and has connectivity with standards such as FHIR.

We evaluated our approach using an end-to-end real case study of an Alzheimer’s patient with a cognitive disability. Although we showed the usefulness of the approach, the main limitations or threats to the validation of MoSIoT are as follows: (i) the need to perform additional case studies to estimate the presented approach’s general applicability; (ii) the need to increase the empirical evidence by conducting experiments to validate the intention to adopt MoSIoT by the physicians who configure the scenarios and by the patients who evaluate the results obtained; (iii) the implementation supporting the MoSIoT proposal was achieved using Azure IoT Central, potentially limiting the commercially available devices for disabilities—to address this problem, different transformations in the simulator should be proposed to have connections to different IoT hubs in the future; and (iv) unlike other proposals such as [14,15,16], which presented highly optimized solutions for specific devices, MoSIoT cannot compete in the search for such results. On the contrary, our approach mainly focuses on adapting the software side of an IoT scenario, delegating to third parties the development of the hardware devices.

Simulation is an initial and necessary step for developing an IoT system, and particularly for an IoT HMS for people with disabilities, which presents considerable complexity and variability. However, the complete system’s materialization is necessary, which implies the implementation of the applications with which people directly interact, according to their type of disability and their different access modes. The MoSIoT framework, through its integration with an IoT hub, provides a robust and extensible architecture for completing the user interfaces and the integration of their devices.

### Future Work

Future investigations are necessary to validate the conclusions drawn from this study. It is necessary to carry out empirical experiments to determine the intention of adoption and the level of satisfaction with the proposed approach, among both medical experts and patients with disabilities. This information will be critical to improve both the interaction with the application and to detect shortcomings in the simulator for enhancement. Furthermore, the current version of MoSIoT allows the definition of scenarios using forms in an app. However, in the future, we would like to provide an interface that allows the instantiation of scenarios using graphical models. It will then be necessary to determine if the graphical models represent an improvement in efficiency and effectiveness compared to the current proposal.
